# The role of enkephalinergic systems in substance use disorders

**DOI:** 10.3389/fnsys.2022.932546

**Published:** 2022-08-05

**Authors:** Lauren G. Rysztak, Emily M. Jutkiewicz

**Affiliations:** ^1^Department of Pharmacology, University of Michigan, Ann Arbor, MI, United States; ^2^Neuroscience Graduate Program, University of Michigan, Ann Arbor, MI, United States

**Keywords:** opioid, enkephalin, substance use disorder, reward, circuitry

## Abstract

Enkephalin, an endogenous opioid peptide, is highly expressed in the reward pathway and may modulate neurotransmission to regulate reward-related behaviors, such as drug-taking and drug-seeking behaviors. Drugs of abuse also directly increase enkephalin in this pathway, yet it is unknown whether or not changes in the enkephalinergic system after drug administration mediate any specific behaviors. The use of animal models of substance use disorders (SUDs) concurrently with pharmacological, genetic, and molecular tools has allowed researchers to directly investigate the role of enkephalin in promoting these behaviors. In this review, we explore neurochemical mechanisms by which enkephalin levels and enkephalin-mediated signaling are altered by drug administration and interrogate the contribution of enkephalin systems to SUDs. Studies manipulating the receptors that enkephalin targets (e.g., mu and delta opioid receptors mainly) implicate the endogenous opioid peptide in drug-induced neuroadaptations and reward-related behaviors; however, further studies will need to confirm the role of enkephalin directly. Overall, these findings suggest that the enkephalinergic system is involved in multiple aspects of SUDs, such as the primary reinforcing properties of drugs, conditioned reinforcing effects, and sensitization. The idea of dopaminergic-opioidergic interactions in these behaviors remains relatively novel and warrants further research. Continuing work to elucidate the role of enkephalin in mediating neurotransmission in reward circuitry driving behaviors related to SUDs remains crucial.

## Introduction

Substance use disorders (SUDs; also known as drug addiction) are characterized by an inability to control drug use, continuing drug use despite adverse consequences, and relapse even after long periods of abstinence. Multiple risk factors contribute to vulnerability for developing of a SUD, such as genetic and environmental factors (for review, see: Volkow and Li, 2005). Due to its chronic relapsing nature, long-term treatment and abstinence is difficult. Research into the neurobiological substrates of SUDs may reveal mechanistic insight into the development of and relapse to SUDs and provide potential targets for therapeutics.

Current theories of the mechanisms underlying SUDs emphasize the role of the mesolimbic dopamine system. “Classic” drugs of abuse, such as psychostimulants, opioids, and nicotine, that maintain self-administration behavior in both animal models and humans, induce a characteristic elevation in dopamine in the nucleus accumbens (NAc) after administration (for review, see: Di Chiara et al., 2004). This can occur *via* stimulation (or disinhibition) of dopamine neurons in the ventral tegmental area (VTA) projecting to the NAc and/or by inhibiting the reuptake of dopamine in the NAc and is thought to be the critical mechanism underlying the primary reinforcing effects of drugs of abuse. Drug-paired cues, one important factor contributing to relapse, can also lead to increased dopamine in the NAc, which further supports other frameworks explaining the role of dopamine in various aspects and stages of SUDs, such as the opponent process and incentive salience theories (for reviews, see: Berridge, 2007; Trigo et al., 2010). In addition to dopamine, numerous neurotransmitter and receptor systems have been implicated in the adaptations caused by drugs of abuse and in the transition from recreational use to SUDs. The endogenous opioid system, comprised of multiple opioid receptor types and endogenous ligands, is highly expressed in reward circuitry and has been proposed to be a crucial modulator of SUDs (for review, see: Trigo et al., 2010).

Positron emission tomography (PET) imaging in human subjects have suggested a potential role of endogenous opioids in the effects of drugs of abuse. For example, oral administration of amphetamine in male subjects reduced binding of [^11^C]carfentanil, a radiolabeled molecule that binds to mu opioid receptors, in the basal ganglia, frontal cortex, and thalamus after amphetamine administration, suggesting that endogenous opioid peptides were released and displaced carfentanil (Colasanti et al., 2012; Mick et al., 2014). Further evidence in human subjects also supports a potential role for endogenous opioid systems in SUDs (Chan et al., 2020). Administration of non-selective opioid receptor antagonists, such as naltrexone or naloxone, may be effective in treating psychostimulant use disorder (Comer et al., 2013) and may reduce cigarette consumption and the satisfaction during *ad libitum* smoking (Covey et al., 1999), although these results are not consistent across all studies (Sutherland et al., 1995). Generally, these reports suggest that the endogenous opioid system plays a role in modulating the effects of drugs of abuse and SUDs, warranting further investigation into the role of opioids.

Most opioid receptor types (mu, delta, kappa, and ORL1) and endogenous opioid peptides (β-endorphin, enkephalins, dynorphins, and others) have been implicated, to some extent, in the neuroadaptations that occur following administration of different drugs of abuse as well as in reward-related behaviors. For many years, each opioid peptide was thought to be primarily selective for one opioid receptor type; however, more recent studies indicate that opioid peptides bind to and activate all three of the canonical opioid receptors, albeit with different affinities and efficacies (Gomes et al., 2020). Previous reports have reviewed the potential role of β-endorphin (Roth-Deri et al., 2008; Le Merrer et al., 2009) or dynorphin (Banks, 2020; Karkhanis and Al-Hasani, 2020; Koob, 2020; Best et al., 2022; Ragu Varman et al., 2022) in SUDs. Therefore, this review will focus specifically on the role of the endogenous enkephalinergic system (e.g., enkephalin peptides and receptors they bind to) in modulating the reward pathway and reward-related behaviors because (1) there is widespread synthesis and release of enkephalins in the reward pathway and (2) the receptor targets of enkephalin are also widely distributed throughout the reward circuitry, namely the mesolimbic and nigrostriatal pathways (for reviews, see Akil et al., 1984; Shippenberg et al., 2008; Le Merrer et al., 2009; Trigo et al., 2010).

It is important to note that studies rarely evaluate the *exclusivity* of enkephalins and enkephalin-induced opioid receptor activation in the neurobiological mechanisms of SUDs. It is also possible that enkephalins always act in conjunction with other opioid peptides and simultaneously at multiple opioid receptor types to produce their effects. Interestingly, there is still much unknown about endogenous enkephalins. In many instances, the sites of enkephalin synthesis and release are not fully appreciated but are thought to be released in response to drugs of abuse and likely play a role in regulating certain behaviors (described below). On the other hand, β-endorphin is synthesized primarily in the arcuate nucleus and nucleus of the solitary tract with fibers projecting to many brain regions, including parts of the reward pathway such as the VTA and NAc, as well as released from the pituitary gland into circulation (Lee and Wardlaw, 2007; Roth-Deri et al., 2008). Therefore, both of these endogenous opioid peptides are likely involved in SUDs and potentially have overlapping, or possibly redundant, roles. For the purposes of this review, we consider the enkephalinergic system to be comprised of enkephalins, enkephalin-hydrolyzing enzymes, and the receptors activated by enkephalins as described below. Hopefully, by combining knowledge from different studies, we will eventually understand the function of endogenous opioidergic systems in reward, motivation, and SUDs.

### Basic biology

There are three primary opioid peptide gene families: proopiomelanocortin (*POMC*), proenkephalin (or preproenkephalin; *PENK*), or prodynorphin (*PDYN*). These genes are translated into prepropeptides (*POMC*, proenkephalin A, and *PDYN*, respectively) before being cleaved into the final functional peptides, β-endorphin, enkephalin, and dynorphin. The primary peptides share a common amino acid N-terminal sequence Tyr-Gly-Gly-Phe-X (Met/Leu for enkephalin). A fourth family of opioid peptide, nociceptin, is derived from prepronociceptin.

Proenkephalin A is cleaved into six copies of met-enkephalin and one copy of leu-enkephalin (Akil et al., 1984; Mclaughlin, 2006). Leu-enkephalin can also be derived from *PDYN* (Akil et al., 1984). Therefore, met-enkephalin may be a more specific marker of proenkephalin activity. Enkephalins are inactivated by two membrane-bound (or soluble) metallopeptidases: neutral endopeptidase (NEP) and aminopeptidase N (APN) (Roques et al., 1980; Ramírez-Sánchez et al., 2019). These peptidases are found near synapses (Ramírez-Sánchez et al., 2019) and are located in brain regions also containing enkephalins, such as the caudate putamen, globus pallidus, substantia nigra, and spinal cord (Waksman et al., 1986). While commonly referred to as enkephalinases, these peptides can also contribute to the formation and degradation of other peptides and/or peptide fragments. While this has brought about the renaming of some of these enzymes, such as enkephalinase to neprilysin (Bayes-Genis et al., 2016), we will refer to enzymes that cleave enkephalin as enkephalinases; however, we recognize that this nomenclature does not include the breadth of activity of these enzymes.

Opioid peptides bind to opioid receptors, which are G-protein coupled receptors (GPCRs). These receptors are coupled to the Gi/o proteins, leading to inhibition of cAMP, inhibition of Ca^2+^ channels, activation of inwardly rectifying K^+^ channels and MAP kinase pathway, which ultimately inhibits neuronal activation and neurotransmitter release (Law et al., 2000). Each receptor is encoded by separate genes, MOR: *Oprm1*, DOR*: Oprd1*, KOR*: Oprk1*, and ORL1*: Oprl1.* Canonically, it is believed that β-endorphin, met-/leu-enkephalin, and dynorphin preferentially bind to the mu opioid receptor (MOR), delta opioid receptor (DOR) and kappa opioid receptor (KOR), respectively. Nociceptin/orphanin FQ binds to the nociceptin opioid peptide receptor [NOPR; or opioid receptor-like 1 (ORL1)]. Enkephalins bind with high affinity to DOR and MOR [with slightly greater affinity (10-fold) for DOR than MOR; measured under non-physiological conditions] (Raynor et al., 1994), but more recently, all opioid peptides have been shown to bind to each of the opioid receptors to some extent (Gomes et al., 2020). For example, β-endorphin, met-enkephalin, and dynorphin have been shown to be full agonists at MOR and partial agonists at DOR. Shorter forms of β-endorphin, generally thought to have limited activity at opioid receptors, are agonists at MOR (Gomes et al., 2020). Therefore, focusing on enkephalin-DOR or enkephalin-MOR interactions in studies investigating SUDs may be overlooking important interactions of other endogenous opioid peptides and receptor types. Overall, while the studies described here implicate enkephalin in multiple aspects of SUDs, there are likely distinct and overlapping roles of other endogenous opioid peptides as well.

### Anatomy & distribution in reward circuitry

Some primary regions of enkephalin release occur within the reward pathway, specifically in the NAc, VTA, and pallidum [comprised of the ventral pallidum (VP) and globus pallidus (GP)]. Interestingly, it is unclear where enkephalin in the NAc comes from, with some studies suggesting that it comes from projection neurons (e.g., dorsal raphe nucleus to NAc shell; Castro et al., 2021) and/or from local release within the NAc (Al-Hasani et al., 2018). On the other hand, the source of enkephalin release in the VP is likely from dopamine D2 receptor-expressing medium spiny neurons (MSNs) in the NAc projecting to the VP (Kalivas et al., 1993; Heinsbroek et al., 2017), but it is unknown whether or not these projections are the only source of enkephalin in the VP. These D2-expressing MSNs projecting from the NAc to the VP presumably release enkephalin as well as GABA, and are considered part of the “indirect” pathway (Zahm et al., 1985), while D1 MSNs (expressing dynorphin) are part of the “direct” pathway, regulating motor function, movement, and reward (Yager et al., 2015). Enkephalin-containing cell bodies seem to be present in the VTA (Johnson et al., 1980; Khachaturian et al., 1983), and are presumably the source of enkephalin release in this brain region, yet this has not been directly tested. Without having a better understanding of sites of enkephalin synthesis and the projection of enkephalin-containing neurons, our knowledge of enkephalinergic circuitry in mediating aspects of SUDs will be limited. Further work needs to be done to better identify the source of enkephalin peptide synthesis and release within the reward circuitry.

Significantly more is known about the expression of both MOR and DOR in reward circuitry. Both opioid receptor types are highly expressed in the same regions with PENK mRNA (Mansour et al., 1993, 1994), including the NAc, caudate putamen, and amygdala. MOR and DOR expression in the mesolimbic circuitry of the rodent brain have been confirmed by autoradiography as well as with expression of fluorescently labeled opioid receptors (GFP-labeled DOR and mCherry-labeled MOR; Erbs et al., 2015). Furthermore, their exact localization on neurons informs us how these receptors regulate neurotransmitter release and/or neuronal activation.

MORs are thought to be located pre- and postsynaptically on neurons in mesolimbic areas (for review, see: Shippenberg et al., 2008) and more specifically on dendrites or dendritic spines in the NAc, amygdala, and VTA near terminals expressing and, presumably, releasing enkephalin (Svingos et al., 1996; Herman et al., 2022). On D2-expressing MSNs, MORs are expressed both postsynaptically in the NAc (Castro and Berridge, 2014) and in the VP, capable of regulating GABA release in the VP (Heinsbroek et al., 2017). Presumably, activation of MORs on D2 MSN terminals in the VP should also regulate enkephalin release; however, this has not been directly tested. MORs have also been identified postsynaptically on pallidal cell bodies (Olive et al., 1997). MORs located in the VTA are present on GABAergic interneurons, such that MOR activation (either *via* exogenous or endogenous ligands) leads to disinhibition of VTA dopamine neurons projecting to the NAc (Johnson and North, 1992).

DORs are thought to be located primarily on axons and axon terminals, on both enkephalin and non-enkephalin releasing neurons (Svingos et al., 1998). In axons and axon terminals, DORs may not always be expressed only on the cell surface, but also located intracellularly and trafficked to the surface under certain conditions (Wang et al., 2008). Within the NAc, DORs have been found on terminals of glutamate neurons projecting from the prefrontal cortex (PFC) (Svingos et al., 1999; Castro and Berridge, 2014; Mongi-Bragato et al., 2018), on cholinergic interneurons (in addition to MORs; Castro and Berridge, 2014; Laurent et al., 2014) and (to a lesser extent) on dopamine terminals (Svingos et al., 1999). Similarly, in the VTA, DORs are expressed presynaptically on GABAergic terminals and can modulate GABA release (Margolis et al., 2008). In contrast, other studies have shown that DORs are expressed postsynaptically on D2 MSNs in the NAc (Castro and Berridge, 2014) and on cell bodies in the VP (Olive et al., 1997).

The presence of enkephalin in the primary cell type of the NAc and prevalence of DORs and MORs throughout the reward pathway further implicates its central role in modulating reward-related neurotransmission. However, the widespread distribution of enkephalin and overlapping MOR and DOR expression in many brain regions and cell types begins to highlight the complexity (and possible redundancy) of the endogenous opioid system in mediating SUDs.

### Methods used to evaluate enkephalin

There are different methods and techniques to evaluate enkephalinergic involvement in reward-related pathways and behaviors. Methods for measuring enkephalin release are limited (for review, see: Conway et al., 2022); therefore, studies often measure enkephalin concentrations in various brain regions as indirect measures of releasable peptide or a releasable pools. Peptide expression and release are likely related, such that if there is increased peptide synthesized, packaged in vesicles, and available for release (intracellular expression), then more peptide is actually released (either tonically or during stimulated release).

Enkephalin peptide concentration can be measured using highly sensitive radioimmunoassay (RIA). Antibodies used in these assays that bind to enkephalin peptide have limitations in selectivity. RIAs with tissue samples also cannot discriminate between intracellular expression and extracellular release of enkephalin. Other, more direct, approaches include collecting dialysate samples *via* microdialysis and then performing RIAs to quantify enkephalin levels in dialysate (first described in Maidment et al., 1989). Enkephalin from microdialysis samples can also be quantified *via* liquid chromatography couple with mass spectrometry (LCMS) and, while technically challenging, can distinguish between met- and leu-enkephalin (Mabrouk et al., 2011; DiFeliceantonio et al., 2012). Methods to selectively activate enkephalin expressing neurons (e.g., optogenetics or designer receptors exclusively activated by designer drugs; DREADDS) can be used to induce the release of enkephalin; however, these methods are not specific to either met- or leu-enkephalin and can also presumably induce the release of other opioid peptides (Al-Hasani et al., 2018) and/or cotransmitters, such as GABA. Therefore, while technical advancements in methodology have allowed for greater specificity in investigating enkephalin, there are still shortcomings that need to be addressed.

In the absence of direct measurements, enkephalin expression and/or levels of enkephalin can be manipulated in order to evaluate the role of enkephalin in SUDs. This has been accomplished through pharmacologically inhibiting enkephalin breakdown or by constitutive global knockout (KO) of the *PENK* gene (and recently conditional knockouts) (for review, see Charbogne et al., 2014). Studies using these tools have provided great insight into the role of the enkephalinergic system in SUDs; however, similar opioid (or non-opioid) peptides and compensatory mechanisms could distort the role of enkephalin specifically. For example, β-endorphin, which has similar affinity at MOR and DOR, may compensate for the lack of enkephalin in KO animals (Maldonado et al., 2018) or leu-enkephalin generated from *PDYN* in PENK KO animals.

Drugs that inhibit enkephalinase, such as thiorphan (Roques et al., 1980) or RB101 (Jutkiewicz, 2007; Jutkiewicz and Roques, 2012), can be used as tools to probe the enkephalinergic system in reward related behaviors by preventing the breakdown of extracellular enkephalin, increasing its activity at MORs and DORs. One limitation of this approach is that there is no way to discriminate between activity due to met- or leu-enkephalin. In addition, these enzymes may also cleave other peptides, such as cholecystokinin (Durieux et al., 1985) and substance P (Matsas et al., 1983); however, studies often perform further experiments to confirm the effects produced by enkephalinase inhibitors occur *via* the activation of opioid receptors. Importantly, β-endorphin has been shown to be a substrate of NEP and APN, but is also degraded by other enzymes (Roques et al., 2012). Many of the opioid receptor-specific behavioral effects of enkephalinase inhibitors (described below) seem to be mediated *via* enkephalins or at least by peptides binding to either MORs or DORs (Noble et al., 2008) because they are blocked by non-selective or selective opioid receptor antagonists. While these tools have been valuable for probing enkephalin peptide in reward related behaviors, there is relatively little is known about enkephalinase activity/mechanisms nor how the enzymes regulate synaptic enkephalin peptide levels. Recent studies have begun to investigate endogenous inhibitors of enkephalinase (Wisner et al., 2006; Tóth et al., 2012) and further investigation into the metabolism of enkephalin *in vivo* (Xu et al., 2010; Wilson et al., 2020) will be crucial for understanding the role of enkephalin in SUDs.

Indirect measurements of enkephalin also provide valuable insight into the enkephalinergic system, albeit with some deficiencies. Quantifying levels of PENK mRNA expression identifies brain regions where enkephalin is likely synthesized, but may not accurately reflect enkephalin peptide expression (intra or extracellular) nor enkephalin release. Similarly, using pharmacological methods to activate or inhibit DOR and/or MOR implicate opioid receptor signaling and requires highly selective ligands. While it is presumed that enkephalin is the endogenous ligand acting on those receptor systems, it is often not directly tested. Since all endogenous opioid peptides bind, to some degree, to all opioid receptors, peptides other than met- or leu-enkephalin may be responsible for the effects measured. Overall, while a wealth of literature has supported the notion that enkephalin modulates reward-related neurobiology and behavior, there is much still to be elucidated.

This review primarily focuses on studies investigating PENK or enkephalin peptides, as they are more closely related to the functional role of enkephalins in reward-related behaviors. Pharmacological studies investigating the effects of DOR and/or MOR activation are not the focus of this review and are thoroughly covered elsewhere (for reviews, see Shippenberg et al., 2008; Le Merrer et al., 2009; Trigo et al., 2010), but some studies are included in this review to extrapolate or corroborate the involvement of enkephalin in modulating reward-related neurotransmission and behaviors.

## Effects of enkephalin on neurotransmission in reward pathways

As described above, studies have attempted to investigate the role of enkephalin in neurotransmission using PENK KO models, increasing levels of enkephalin by preventing breakdown, and activation of enkephalins’ targets with exogenous ligands. By using these approaches enkephalins have been identified as neuromodulators, influencing release and extracellular levels of dopamine, GABA, glutamate, acetylcholine, and other neurotransmitters involved or implicated in reward-related circuits (for review, see Torregrossa and Kalivas, 2008).

### Enkephalinergic modulation of dopamine neurotransmission

The most prevalent mechanisms underlying SUDs center around the role of dopamine in driving drug-taking and -seeking behaviors, and there is strong evidence of interactions between enkephalinergic and dopaminergic systems. Perhaps the most obvious interaction between the two systems is that MOR activation by exogenously administered agonists, such as morphine, stimulate dopamine release in the NAc. Further, KOR activation reduces dopamine release (Escobar et al., 2020), and DOR activation may increase dopamine release to some extent (Saigusa et al., 2017) or have no effect on dopamine (Longoni et al., 1998).

To further elucidate the role of enkephalin on the dopaminergic system, studies have measured dopamine neurotransmission in PENK KO mice. Basal levels of dopamine in the NAc did not differ between PENK KO and wild-type animals (Berrendero et al., 2005), but evoked-dopamine levels appear to be altered by enkephalin and opioid receptor activation. For example, a dose of nicotine that stimulates dopamine release in wildtype mice had a blunted dopamine response in the NAc in PENK KO mice (Berrendero et al., 2005). To our knowledge, no other effects of drugs of abuse on dopamine levels in the NAc of PENK KO animals have been reported. It is possible that enkephalin promotes nicotine-stimulated dopamine release, likely *via* opioid receptor-induced inhibition of GABA release in the NAc and/or VTA.

**FIGURE 1:**
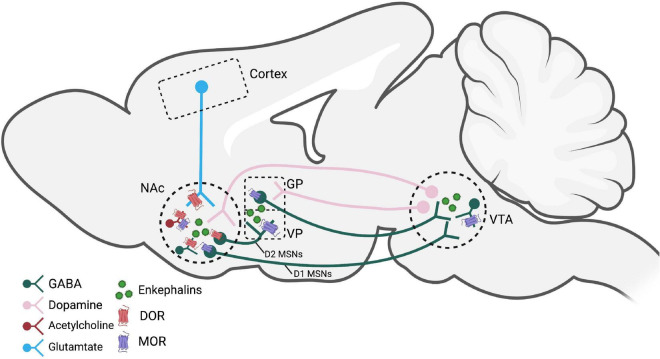
Brain regions and pathways implicated in enkephalin-mediated reward-related behaviors. Dopamine neurons in the VTA that project to the NAc are modified by MORs on GABAergic interneurons. Activation of MORs and DORs, likely by enkephalins, within the NAc modulate dopamine, GABA, glutamate, and acetylcholine release. D2 MSNs express enkephalin and project to the VP and are believed to be a crucial circuit for reinstatement behaviors. Figure created using Biorender.com. NAc, nucleus accumbens; GP, globus pallidus; VP, ventral pallidum; VTA, ventral tegmental area; MOR, mu opioid receptor; DOR, delta opioid receptor; MSNs, medium spiny neurons.

Consistent with the study described above, opioid receptor activation also enhances psychostimulant-induced increases extracellular levels of dopamine in the NAc (see Figure 1). For example, increasing endogenous enkephalins by blocking hydrolysis with an enkephalinase inhibitor, thiorphan, given into the substantia nigra potentiated amphetamine-stimulated dopamine release in the striatum (Schad et al., 2002). Conversely, preventing activation of opioid receptors on inhibitory GABAergic neurons locally in the substantia nigra, VTA, or GP attenuated amphetamine-induced increases in dopamine in their projection targets, the striatum, NAc, and locally in the GP, respectively (Schad et al., 1995, 2002; Mabrouk et al., 2011). In the absence of other drugs, naloxone given locally into the GP decreased dopamine in the same brain region, suggesting that there is a tonic enkephalinergic tone in the GP which activates MORs (presumably) on GABAergic terminals to inhibit GABA release and ultimately disinhibit dopamine (Mabrouk et al., 2011).

Enkephalin binds to and activates MOR and DOR; therefore, exogenous administration of MOR and DOR agonists have been used to probe the potential involvement (albeit indirectly) of endogenous enkephalins in regulating dopamine neurotransmission. MOR agonists increase dopamine in the dorsal and ventral striatum (*via* disinhibition) by activating MORs in the VTA/substantia nigra. MORs do not seem to be located presynaptically on dopamine terminals in the NAc (Svingos et al., 1996; Britt and McGehee, 2008; Saigusa et al., 2017; but see Svingos et al., 1999), but may be present presynaptically in the VP to gate dopamine release arising from the VTA (Mitrovic and Napier, 2002; Root et al., 2015; Clark and Bracci, 2018).

While the effects of MOR activation on dopaminergic neurotransmission are fairly well-explored, the effects of DOR activation on dopamine levels are unclear. For example, the peptide DPDPE given intracerebroventricularly dose-dependently increased dopamine in the NAc of anesthetized rats, which was blocked by the DOR antagonist ICI 174,864 (Spanagel et al., 1990). Also, the small molecule DOR agonist SNC80 failed to promote dopamine efflux in rat striatal preparations directly (Bosse et al., 2008) and failed to increase dopamine levels in the NAc or caudate putamen in rats measured by microdialysis (Longoni et al., 1998). However, SNC80 did enhance amphetamine-mediated dopamine efflux in the striatum (Bosse et al., 2008) as well as amphetamine-mediated locomotor activity (Jutkiewicz et al., 2008) potentially through indirect actions with glutamatergic neurons. The effects of DOR activation on modulating dopaminergic neurotransmission is unclear (for review, see: Saigusa et al., 2017), but overall, enkephalin and opioid receptor activation seem to have some neuromodulatory effects on dopaminergic activity in the reward pathway, most likely through indirect mechanisms.

Together, these findings indicate that endogenous enkephalins in the VTA, substantia nigra, and potentially other brain regions may contribute to drug-induced increases in dopamine in the NAc. These studies indicate that opioid receptor activation enhances dopamine, likely *via* disinhibition. Extrapolating from these indirect studies of opioid receptor activation, it is plausible to think that endogenous activation of these same opioid receptors would be able to enhance dopamine reward circuitry and potentiate SUDs.

### Enkephalinergic modulation of GABA neurotransmission

As described above, opioid-induced inhibition of GABAergic neurons in the VTA and substantia nigra (Galaj et al., 2020; Oliver, 2021) disinhibits dopamine neurons projecting to the NAc (Johnson and North, 1992). Thus, opioids have been shown to regulate GABA release within the reward pathway. GABA and enkephalin are thought to be cotransmitters, released from D2 MSNs projecting from the NAc to the ventral pallidum (Maneuf et al., 1994) where enkephalin likely binds to presynaptic opioid receptors (or autoreceptors) to inhibit further GABA and enkephalin release (Maneuf et al., 1994; Stanford and Cooper, 1999). Indeed, in VP slices prepared from drug naïve rats, the administration of an enkephalinase inhibitor reduced extracellular levels of GABA in the VP (Kupchik et al., 2014). Whereas naloxone given locally into the pallidum, increased GABA and also decreased dopamine in the same brain region (Mabrouk et al., 2011). At least some studies have suggested that MORs are located on GABAergic terminals in the VP and VTA, such that exogenous activation of MORs inhibits GABA release in the VP (Kalivas et al., 2001) and in the VTA (Matsui and Williams, 2011; Matsui et al., 2014).

Additionally, activation of DORs likely influences GABAergic transmission in reward circuitry, but this has not been investigated thoroughly. Within the NAc, activation of DORs present on GABAergic terminals reduce inhibitory neurotransmission (Jiang and North, 1992; Chieng and Williams, 1998). In the VTA, postsynaptic DOR activation has been shown to augment GABA_*A*_ receptor mediated inhibitory postsynaptic currents (Margolis et al., 2011). Overall, these studies suggest that enkephalins primarily act on GABAergic terminals or interneurons to inhibit GABA release in multiple brain regions.

### Enkephalinergic modulation of glutamate neurotransmission

Glutamate, the primary excitatory neurotransmitter in the brain, has been shown to drive reward-related behaviors, such as sensitization and reinstatement (Scofield et al., 2016). The VTA receives glutamatergic projections from multiple brain regions (Geisler et al., 2007; Watabe-Uchida et al., 2012); however, it is unclear whether opioids influence glutamate neurotransmission in the VTA. There is evidence in other brain regions that opioids can modulate glutamate neurotransmission. For example, glutamatergic neurons project from the amygdala to the VP and release can be inhibited *via* MOR agonists (Mitrovic and Napier, 1998). VP glutamatergic neurons are preferentially innervated by D1 MSNs arising from the NAc (Heinsbroek et al., 2020), therefore there is likely opioid modulation of glutamatergic activity within the VP *via* dynorphin release, but this has not been directly tested.

Glutamate release in the NAc stems from projection neurons originating in the prefrontal cortex and enhances dopamine release in the NAc (Tzschentke and Schmidt, 2003). Glutamatergic axon terminals in the NAc express opioid receptors, specifically DOR (Winters et al., 2017; see Figure 1). Therefore, activation of DOR, by enkephalins, on the terminals of PFC-projecting glutamatergic neurons would be likely to decrease glutamate release in the NAc. However, DORs are not expressed exclusively on glutamatergic terminals in the NAc, highlighting the complexity of the endogenous opioid system in this brain region and how it might influence glutamatergic neurotransmission. For example, the DOR agonist, SNC80, has been shown to indirectly increase glutamate efflux in the striatum (Bosse et al., 2014). The proposed mechanism is that SNC80 activates DOR on GABAergic terminals, thereby inhibiting GABA release, which leads to local glutamate release and, subsequently, potentiation of amphetamine-induced dopamine release. An NMDA receptor antagonist, MK801, blocked the effects of SNC80 on enhancing dopamine (Bosse et al., 2014), suggesting that DOR activation can modulate the excitatory/inhibitory balance within the striatum to disinhibit dopamine release. Consistently, local administration of naltrindole, a DOR antagonist, into the caudate putamen blocked amphetamine-induced increases in glutamate, which was reversed by the DOR agonist, DPDPE (Rawls and McGinty, 2000).

Interestingly, there is some evidence that opioid receptors are also located on glia in the NAc, potentially suggesting a regulatory role of enkephalin on non-neuronal glutamate neurotransmission (Corkrum et al., 2019). Together, these studies suggest endogenous enkephalin acts as a direct or indirect neuromodulator of glutamate neurotransmission and may modulate changes in glutamate neurotransmission induced by drugs of abuse. However, the role of enkephalin has not been evaluated directly.

### Enkephalinergic modulation of cholinergic neurotransmission

Cholinergic interneurons also have an important function in regulating neurotransmission in reward centers. In the NAc, cholinergic interneurons are the only source of acetylcholine and act locally to regulate efferents, particularly glutamate and dopamine (Warner-Schmidt et al., 2012). Specifically, MOR and DOR expression on cholinergic interneurons indicates that endogenous opioid peptides ligands may act as neuromodulators of acetylcholine release (Laurent et al., 2014; see Figure 1). Indeed, DOR and MOR activation by leu-enkephalin or DAMGO decreased acetylcholine release in the striatum (Mulder et al., 1984; Jabourian et al., 2005; Arttamangkul et al., 2021). While cholinergic neurons are also present in both the GP/VP (Chiba et al., 1995) and VTA (Rada et al., 2000; Mathon et al., 2003), it is unclear if or how endogenous opioid peptides modulate cholinergic release or signaling in these brain regions. Therefore, enkephalin may have an additional role of regulating cholinergic inhibition in brain regions within the reward pathway.

Overall, enkephalins acting at MORs or DORs modulates the transmission of multiple neurotransmitter systems enhancing the reward-related circuitry through inhibition of GABA or disinhibition of glutamate and/or dopamine. It is important to note that many of the described studies extrapolate from indirect measures of the involvement of enkephalins, because enkephalins are rarely measured directly.

## Drugs alter enkephalin levels: Peptide levels and mRNA

There is also evidence that drugs of abuse may increase enkephalin release by unknown mechanisms, stimulating MORs and DORs, further potentiating extracellular levels of glutamate and dopamine (by mechanisms described above) and thus driving reward-related behaviors. Acute and chronic administration of drugs of abuse have been shown to alter levels of enkephalin in reward brain regions, albeit with some inconsistent results across studies.

### Indirect and direct dopamine receptor agonists

It has been shown that amphetamine administration increases enkephalins in multiple brain regions. Amphetamine increased met-enkephalin release in NAc and PFC (Assis et al., 2006, 2009), and in the GP (Mabrouk et al., 2011). Similarly, cocaine administration caused displacement of radioactive DAMGO at MORs in the NAc, suggesting that cocaine may stimulate endogenous opioid release, potentially enkephalins, β-endorphin, or other opioid peptides (Roth-Deri et al., 2003; Soderman and Unterwald, 2009). However, cocaine did not alter met-enkephalin in striatum or substantia nigra as measured by RIA (Sivam, 1989).

Psychostimulant administration may also alter the expression of endogenous opioid peptide mRNA, which may influence enkephalin levels and release, but there are mixed results of psychostimulant-induced changes in expression of PENK mRNA throughout reward circuitry. These may be due to differences in psychostimulant dose, time of mRNA measurement, and acute versus chronic administration. For example, psychomotor stimulants either increased, did not change, or decreased PENK mRNA in the striatum (Hurd and Herkenham, 1992; Wang and McGinty, 1996; Adams et al., 2000), decreased or did not alter PENK mRNA in the NAc (Adams et al., 2000; Turchan et al., 2002), and did not alter PENK mRNA expression in the amygdala (Turchan et al., 2002). Similar inconsistent results have been reported as a result of cocaine administration. Experimenter-administered repeated cocaine did not alter PENK mRNA in the amygdala, dorsal striatum, NAc shell or core (Mathieu-Kia and Besson, 1998; Turchan et al., 2002), but “binge” and contingent cocaine administration increased PENK mRNA in NAc, caudate putamen, PFC, and substantia nigra (Hurd and Herkenham, 1992; Spangler et al., 1997; Crespo et al., 2001; Mantsch et al., 2004; Sun et al., 2020) but not in the dorsal or ventral striatum (Hurd and Herkenham, 1992; Arroyo et al., 2000). Perhaps, these results suggest that repeated administration of psychomotor stimulants is more likely than acute drug treatment to induce changes in PENK mRNA, suggesting the involvement of long-lasting neuroadaptations as a consequence of chronic drug exposure. While few studies have investigated the effects of psychostimulants on enkephalin peptide levels or release, these limited data suggest psychostimulants may increase enkephalins in certain mesolimbic brain regions, perhaps with some differences between amphetamine and cocaine.

### Opioids

Although enkephalins are an endogenous ligand for MORs, few studies have investigated the effects of exogenous MOR activation on enkephalin levels. Acute morphine (Olive et al., 1995) and heroin (Olive and Maidment, 1998) increased extracellular opioid peptides in the VP/GP thought to be enkephalin, but morphine did not alter enkephalin levels in the NAc (Olive et al., 1995). Repeated morphine was shown to either not alter (Uhl et al., 1988) or increase met-enkephalin (Nylander et al., 1995) in the striatum, NAc, and PAG (Nieto et al., 2002). Similarly, in rats with a history of heroin self-administration, MOR agonists also elevated levels of met- and leu-enkephalin in the caudal striatum and septum (Cappendijk et al., 1999). Morphine conditioning also induced an increase in enkephalin in the NAc (Nieto et al., 2002). Together, these findings suggest that exogenously administered opioids increase enkephalin in the reward pathway and may be involved in the formation of opioid-context associations.

There are few studies assessing the administration of exogenous opioids on PENK mRNA levels. Acute morphine did not alter PENK mRNA in NAc nor striatum (Turchan et al., 1997) and repeated morphine reduced PENK mRNA in NAc (Turchan et al., 1997) and striatum (Uhl et al., 1988). Morphine self-administration reduced PENK in NAc core and shell of LEW rats (Sánchez-Cardoso et al., 2007). These effects on PENK mRNA expression following chronic opioid agonist administration only evaluate enkephalin levels indirectly and are distinctly different from those found following repeated psychostimulant administration.

Together, these findings suggest that, while acute and chronic administration of MOR agonists may increase enkephalin release and peptide levels, chronic opioid administration mainly leads to a reduction in PENK mRNA expression, potentially compensating for the replacement of endogenous opioid peptides by exogenous opioid receptor ligands.

### Ethanol

The effects of ethanol on enkephalin levels and PENK mRNA are highly varied across studies. Acute ethanol has been shown to increase met-enkephalin in the NAc shell and striatum, decrease enkephalin in striatum, hypothalamus, and midbrain and not alter enkephalin in VTA, amygdala, hypothalamus, midbrain, brainstem, and hippocampus (Schulz et al., 1980; Seizinger et al., 1983; Marinelli et al., 2005; Lam et al., 2008; Jarjour et al., 2009; Méndez et al., 2010). It has been hypothesized that acute ethanol may influence enkephalin biosynthesis and release in mesolimbic areas as well (Méndez et al., 2010). Similarly, chronic ethanol increased met-enkephalin in the PAG, decreased met-enkephalin in striatum, hippocampus, brainstem, and midbrain (Schulz et al., 1980; Lindholm et al., 2000) and hypothalamus or was ineffective in altering levels in midbrain and hippocampus (Seizinger et al., 1983).

Ethanol exposure also produces varied changes in PENK mRNA levels in various brain regions. Acute ethanol treatment and voluntary consumption increased PENK mRNA in the paraventricular nucleus of thalamus, caudate putamen, amygdala, PFC, and NAc core and shell (de Gortari et al., 2000; Cowen and Lawrence, 2001; Oliva et al., 2008) and decreased PENK mRNA levels in VTA and NAc (Méndez and Morales-Mulia, 2006) in rats. Ethanol-induced changes in PENK mRNA may reflect phenotypic differences in ethanol preference, as acute ethanol increased PENK mRNA in NAc of alcohol-preferring but not alcohol-non-preferring rats (Li et al., 1998). Despite varying results of ethanol administration on enkephalin peptide and PENK mRNA levels, these studies suggest ethanol has some influence on enkephalin expression that may be brain region dependent, and further work is warranted to continue to parse apart specific effects of ethanol on the enkephalinergic system.

### Nicotine

Few studies have investigated the effects of nicotine administration on endogenous enkephalin peptide levels. Acute and repeated administration of nicotine increase met-enkephalin levels in the striatum of mice as measured by immunoreactivity (Pierzchala et al., 1987; Dhatt et al., 1995; Wewers et al., 1999), and these effects were blocked by a nicotinic acetylcholine receptor antagonist (Dhatt et al., 1995). In human PET studies, nicotine smoking decreased [^11^C] carfentanil binding in certain brain regions, such as prefrontal cortices and ventral striatum (Domino et al., 2015), further suggesting that nicotine administration increases enkephalin release in reward brain regions.

Similar to peptide levels, acute administration of nicotine in mice and rats increased PENK mRNA in the striatum and hippocampus (Dhatt et al., 1995; Houdi et al., 1998). These effects were blocked by the nicotinic acetylcholine receptor antagonist mecamylamine, but not the muscarinic antagonist atropine nor dopamine receptor antagonist haloperidol (Dhatt et al., 1995). The effects of repeated nicotine administration on PENK mRNA also vary across studies and across brain regions (Höllt and Horn, 1992; Dhatt et al., 1995; Houdi et al., 1998; Mathieu-Kia and Besson, 1998; Ugur et al., 2017). Therefore, potential compensatory adaptations in PENK mRNA following repeated nicotine may be different across reward circuitry.

### Cannabinoids

Endogenous cannabinoids and their receptors (CB1) are present in many of the same brain regions as opioid receptors (Befort, 2015), indicating possible overlap and interaction between the two systems. Indeed, acute, moderate doses of THC increased enkephalin-like material in the NAc determined by RIA (Valverde et al., 2001) and increased met-enkephalin immunoreactivity in preoptic area and medial basal hypothalamus after repeated THC exposure (Patel et al., 1985).

The effects of cannabinoids on enkephalins may be greater in non-reward brain regions. Subchronic THC increased PENK mRNA levels in rat hypothalamus, PAG, and mammillary nucleus (Corchero et al., 1997; Manzanares et al., 1998), with no change in the striatum or NAc. Repeated treatment of a synthetic cannabinoid receptor agonist, CP-55,940, also increased PENK mRNA in hypothalamus and additionally the striatum and NAc (Manzanares et al., 1998). Clearly, the effects of cannabinoids on endogenous opioids and PENK mRNA are largely unknown and should be investigated further.

### Other and summary

Other conditions have also been shown to change levels of enkephalin peptides. Consumption of palatable food leads to a surge of met- and leu-enkephalin in the anteromedial portion of the dorsal neostriatum, analyzed by LCMS (DiFeliceantonio et al., 2012). Optogenetic stimulation of dynorphin-expressing neurons in either ventral or dorsal NAc shell leads to increased met- and leu-enkephalin in both brain regions. This could suggest that cross-modulation of opioid peptides occurs within local circuitry in the NAc (Al-Hasani et al., 2018). Together, all of these data suggest that many drugs of abuse (and potentially non-drug reinforcers) increase enkephalin levels, which may underlie and contribute to their reinforcing effects and abuse potential by further promoting reward neurotransmission through inhibition of GABAergic signaling.

## Enkephalinergic system and reward-related behaviors

The role of the endogenous enkephalinergic system has been evaluated in reward related behaviors as well as other potentially related (and co-morbid) behaviors and physiological functions, such as stress resiliency, pain, and emotion (Jutkiewicz and Roques, 2012; Henry et al., 2017; Corder et al., 2018).

For example, increasing enkephalin levels with thiorphan in the VTA (Glimcher et al., 1984) or mimicking enkephalin with a met-enkephalin peptide analog (Phillips and LePiane, 1982) given into the VTA induces conditioned place preference (CPP), in a naloxone-sensitive manner. In addition, infusions of met-enkephalin into the NAc maintained lever pressing behavior (e.g., self-administration behavior), and this behavior was blocked by naloxone (Goeders et al., 1984). Furthermore, preventing the breakdown of endogenous enkephalins with the enkephalinase inhibitor thiorphan increased ethanol intake (Froehlich et al., 1991). These studies indicate that enkephalin may have some primary reinforcing properties and is able to activate reward circuitry.

The rewarding and reinforcing effects of various drugs of abuse are also altered by attenuating endogenous enkephalin signaling with the administration of opioid receptor antagonists or by genetic deletion of PENK. Administration of opioid receptor antagonists, which presumably block the effects of endogenous enkephalins or other opioid peptides, attenuated or blunted cocaine-induced CPP (Menkens et al., 1992), heroin self-administration (Martin et al., 2000; Tomasiewicz et al., 2012), and alcohol seeking behavior and alcohol withdrawal (Perry and McNally, 2013; Alongkronrusmee et al., 2016). Consistently, PENK KO decreased cocaine self-administration (Gutiérrez-Cuesta et al., 2014), cocaine-induced locomotor sensitization (Mongi-Bragato et al., 2016, 2021), and nicotine-induced CPP (Berrendero et al., 2005). However, PENK KO did not alter morphine (Le Merrer et al., 2011) or ethanol self-administration (Koenig and Olive, 2002; Hayward et al., 2004; Racz et al., 2008) or morphine CPP (Skoubis et al., 2005). These studies suggest enkephalinergic signaling, *via* opioid receptor activation, contributes to the rewarding effects of various drugs of abuse. This is likely due to multiple indirect mechanisms culminating in disinhibition of dopamine, either *via* disinhibiting glutamate efferents in NAc or inhibiting GABAergic interneurons in the VTA.

Interestingly, in animals trained to discriminate morphine, systemic administration of an enkephalinase inhibitor, RB 120, did not generalize to the discriminative stimulus effects of morphine and, conversely, morphine did not generalize to the discriminative stimulus effects of RB 120. Together, these data suggest that, even though enkephalins may have some rewarding properties, endogenous enkephalin and the MOR agonists may produce different subjective effects (Hutcheson et al., 2000). Therefore, targeting the endogenous enkephalinergic system for various therapeutic endpoints may lack the abuse liability of high affinity, efficacious MOR agonists. Future studies would need to investigate this further.

The endogenous enkephalinergic system may also be involved in other aspects related to the development and maintenance of SUDs. For example, MOR activation in the NAc and VP enhances hedonic impact or “liking,” a distinct but related function to drug “wanting” (for reviews, see: Smith et al., 2009; Castro and Berridge, 2014). Other evidence suggests enkephalin is involved in the formation of drug-context/cue associations. Activation of MORs or DORs (specific localization unknown) by protected endogenous enkephalins in the NAc or with exogenous agonists induces reinstatement of cocaine-seeking behavior (Simmons and Self, 2009), which was blocked by a MOR antagonist given into the NAc (Simmons and Self, 2009) and VP (Tang et al., 2005) and a DOR antagonist in the NAc. Studies have also shown that cue-induced reinstatement may be a result of cocaine-induced increased enkephalinergic tone in the VP on presynaptic MORs, causing disinhibition of VP neurons projecting to VTA or other brain regions (Heinsbroek et al., 2017, 2020). These interpretations are supported by other findings demonstrating that opioid receptor blockade and MOR and DOR knockout reduced cue-induced cocaine seeking behavior and impaired morphine CPP (Burattini et al., 2008; Gutiérrez-Cuesta et al., 2014). Overall, the enkephalinergic system may act as a modulator of SUD-related behaviors by promoting drug-cue associations that enhance the rewarding effects of drugs of abuse and/or drive drug-seeking behaviors.

## Conclusion

This review highlights the role of enkephalins as neuromodulators of reward-related circuitry and behaviors underlying SUDs. However, many questions still remain. As mentioned earlier, few studies directly identify and measure the specific opioid peptides involved in reward-related neurotransmission and behaviors. Therefore, in many cases, the effects are assumed to be regulated by endogenous enkephalins or other opioid peptides, such as β-endorphin. Further work identifying the specific opioid peptides and their targets (either specific or non-specific receptor targets) will provide a better understanding of the mechanisms involved in SUDs. In order to accomplish this, we must also have an improved appreciation of the sites of enkephalin synthesis, the sources of enkephalins, and the regulation of enkephalin catabolism. Finally, manipulating enkephalin directly and with brain region or cell type specificity will be crucial to measure enkephalinergic influence on reward-related behaviors.

The studies described in this review used a multitude of techniques to probe the role of enkephalin, and each technique has limitations that can influence interpretations of results. Limitations of enkephalin measurement techniques (Conway et al., 2022) are due, in part, to the complexity of the endogenous enkephalinergic system. Opioid peptides are highly homologous peptides that are rapidly degraded and bind to multiple opioid receptor types. Endogenous enkephalins are also released in smaller amounts than “classical” neurotransmitters, complicating measuring techniques. Cleavage of the opioid prepropeptides yield differential, yet overlapping, quantities of each peptide. Again, many of the studies implicating endogenous opioid release may presumably involve enkephalin due to its high prevalence in reward circuitry, yet β-endorphin cannot be ruled out as the ligand or one of the peptides involved.

Technological advancements to improve detection and quantification of endogenous opioid peptides and their regulation by enkephalinases will help our understanding of the role of enkephalins in circuitry and reward-related behaviors. Tools for measuring extracellular enkephalin specifically, such as liquid chromatography coupled with mass spectrometry analysis of *in vivo* samples (Mabrouk et al., 2011; DiFeliceantonio et al., 2012; Al-Hasani et al., 2018) and voltammetry to measure met-enkephalin (Calhoun et al., 2019) can be further applied during drug self-administration and while measuring other reward-related behaviors. Recent advancements in sensors to track dynamics of dopamine can ideally be applied to other neuromodulators like enkephalin (Patriarchi et al., 2018). Similarly, fluorescent reporters that can detect MOR activation are in development (Kroning and Wang, 2021). The ability to measure the dynamics of enkephalin degrading enzymes will also be necessary for better understanding of enkephalin regulation. Other tools such as conditional PENK knockout animal models (Gaveriaux-Ruff et al., 2011; Charbogne et al., 2014), caged-opioids, and allosteric modulators may be further implemented to study endogenous enkephalin release and function. Novel tools for more specific functional manipulations may be better for establishing causality, such as the use of CRISPR-Cas9 technology to selectively knockout enkephalin in specific cell types (Castro et al., 2021).

The studies discussed in this review provide strong evidence that the endogenous enkephalinergic system plays an important role in modulating reward circuitry and driving maladaptive behaviors to SUDs. In order to further understand the underlying mechanisms of SUDs, more research should probe the direct involvement of enkephalins and other opioid peptides in the formation, persistence, and relapse to SUDs. Furthermore, the endogenous enkephalinergic system may also be a potential target for novel therapeutics to prevent and treat SUDs and relapse.

## Author contributions

LR and EJ contributed to the writing and revisions of this review. Both authors contributed to the article and approved the submitted version.
